# Osteochondral Allograft Transplantation for Treatment of a Focal Tibial Plateau Cartilage Lesion

**DOI:** 10.1016/j.eats.2025.103569

**Published:** 2025-05-22

**Authors:** Sebastian Schmidt, Chilan B.G. Leite, Domenico Franco, Alexander Bumberger, Nathan Sherman, Omar Protzuk, Christian Lattermann

**Affiliations:** a Department of Orthopedic Surgery, Brigham and Women’s Hospital, Harvard Medical School, Boston, Massachusetts, U.S.A.; b Department of Orthopaedic and Trauma Surgery, University Medical Centre Mannheim, Medical Faculty Mannheim, University of Heidelberg, Mannheim, Germany; c Department of Orthopedics and Trauma Surgery, University Hospital Vienna, Medical University of Vienna, Vienna, Austria; d Operative Research Unit of Orthopaedic and Trauma Surgery, Fondazione Policlinico Universitario Campus Bio-Medico, Rome, Italy

## Abstract

Focal cartilage lesions of the tibial plateau present unique challenges due to limited regenerative potential and complex biomechanical demands. This Technical Note outlines an osteochondral allograft transplantation technique for treating isolated central defects of the lateral tibial plateau. Fresh donor allografts are precisely matched and prepared, with careful debridement and socket creation to ensure a stable press-fit implantation, secured with bioresorbable nails. The described approach emphasizes technical precision and patient selection. This method offers a targeted and effective solution for central tibial plateau lesions, addressing an unmet clinical need while preserving knee function and structural integrity.

Damage to the articular cartilage is found in up to 65% of all patients who undergo knee arthroscopy.^1^ Hyaline cartilage has limited self-regenerative properties, and if left untreated, cartilage defects can result in progressive joint degeneration, chronic pain, and functional impairment.^2^^,^^3^ Symptomatic cartilage defects are challenging to treat and can arise from trauma, mechanical axis malalignment, meniscal deficiency, ligament insufficiency, or vascular compromise.^4^ Cartilage lesions of the femur and patella articular surfaces are more common than tibial defects, and a wide range of surgical treatments are available.^1^^,^^5^^,^^6^

Treatment options for focal cartilage defects include autologous chondrocyte implantation (ACI) and matrix-induced ACI (MACI), as well as osteochondral autograft transplantation (OAT) and osteochondral allograft (OCA) implantation, among others.^6^ Systematic reviews demonstrate that OAT, OCA, ACI, and MACI all yield significant improvements in patient-reported outcome measurements (PROMs) with low complication rates in patellar and femoral cartilage defects.^6^^,^^7^ They found OAT to be effective for smaller cartilage defects, particularly those less than 2 cm^2^, and also highlighted the effectiveness of OCA, ACI, and MACI for larger defects.^2^, ^7^

There are limited data supporting the use of these modalities in the treatment of cartilage lesions isolated to the tibial plateau (TP).^5^ Surgical treatment of TP cartilage lesions presents distinct challenges, mainly related to obtaining sufficient exposure.^8^ In a recent systematic review, 5 studies including 138 patients were reviewed, and all lesions were unicondylar, requiring an OCA transplant of an entire hemi-tibial plateau.^5^ While most patients had post-traumatic changes and concomitant procedures, such as meniscal transplants, for patients with focal central cartilage damage in the TP area, complete replacement with a hemi-plateau allograft may not be appropriate. While technically demanding, focal OCA transplantation may provide a viable alternative for smaller defects. Here, we describe our technique for an OCA transplantation to the lateral tibial plateau (LTP) to treat a central focal defect.

### Evaluation

The clinical presentation of cartilage lesions of the TP can vary but often includes knee pain, typically localized to the joint line, and worsened by weightbearing activities such as walking, climbing stairs, or prolonged standing. Other symptoms may include joint swelling, tenderness over the TP, restricted range of motion, crepitus along the jointlines, and sensations of instability of the knee. Imaging, especially magnetic resonance imaging, plays a critical role in assessing the extent of cartilage damage and identifying concomitant pathologies such as meniscus lesions. In addition, x-rays are essential to assess the joint under load, to assess signs of osteoarthritis such as osteophytes, and to evaluate for negative prognostic factors such as coronal or sagittal mechanical axis malalignment or deformity. Indications for LTP OCA include failure of nonoperative management or failure of previous regenerative cartilage procedure.

Contraindications for OCA to the LTP include untreated coronal or sagittal malalignment, knee instability, meniscal insufficiency, and tricompartmental cartilage disease. Patients should not have a history of inflammatory arthritis or an active infection. Morbid obesity and smoking are considered relative contraindications for this procedure.

## Surgical Technique

### Osteochondral Allograft Selection

Fresh OCAs are sized preoperatively using magnetic resonance imaging or radiographs to match donor sizing to the host. We utilize an allograft provided by Joint Restoration Foundation.

### Patient Position

For TP cartilage defects requiring knee hyperflexion, the patient is positioned supine on a flat surgical table (Video 1). Otherwise, a leg holder may be used to support the affected limb. A tourniquet is then applied very proximally to the ipsilateral thigh.

### Osteochondral Allograft Transplantation

**Fig 1:**
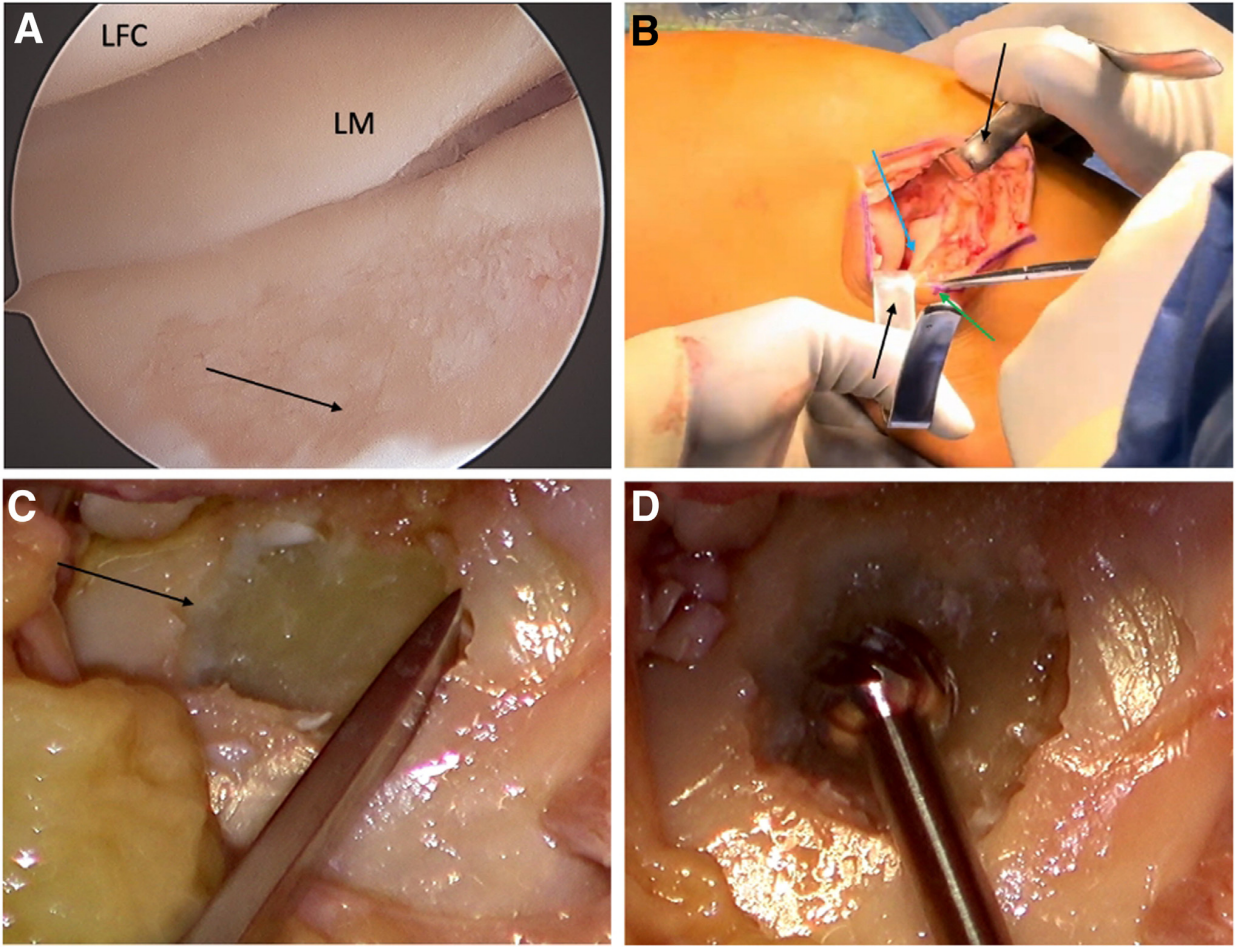
Preparation of osteochondral allograft transplantation to the right knee in flexion. (A) Arthroscopic visualization through the medial portal of the lateral compartment with the lateral femoral condyle (LFC), the lateral meniscus (LM), and the tibial cartilage defect (black arrow). After arthroscopic assessment, the defect is debrided. (B) To expose the defect (blue arrow) a lateral parapatellar arthrotomy is performed. 2 Z-retractors are utilized to optimize direct visualization (black arrows), and the anterior horn of the LM is detached (green arrow). Under direct view through the lateral arthrotomy the tibia plateau defect and with the knee in 70° flexion, varus stress and the tibia internally rotated, the defect is chiseled out rectangularly (C) and debrided with a burr (D) to create a stable socket.

**Fig 2:**
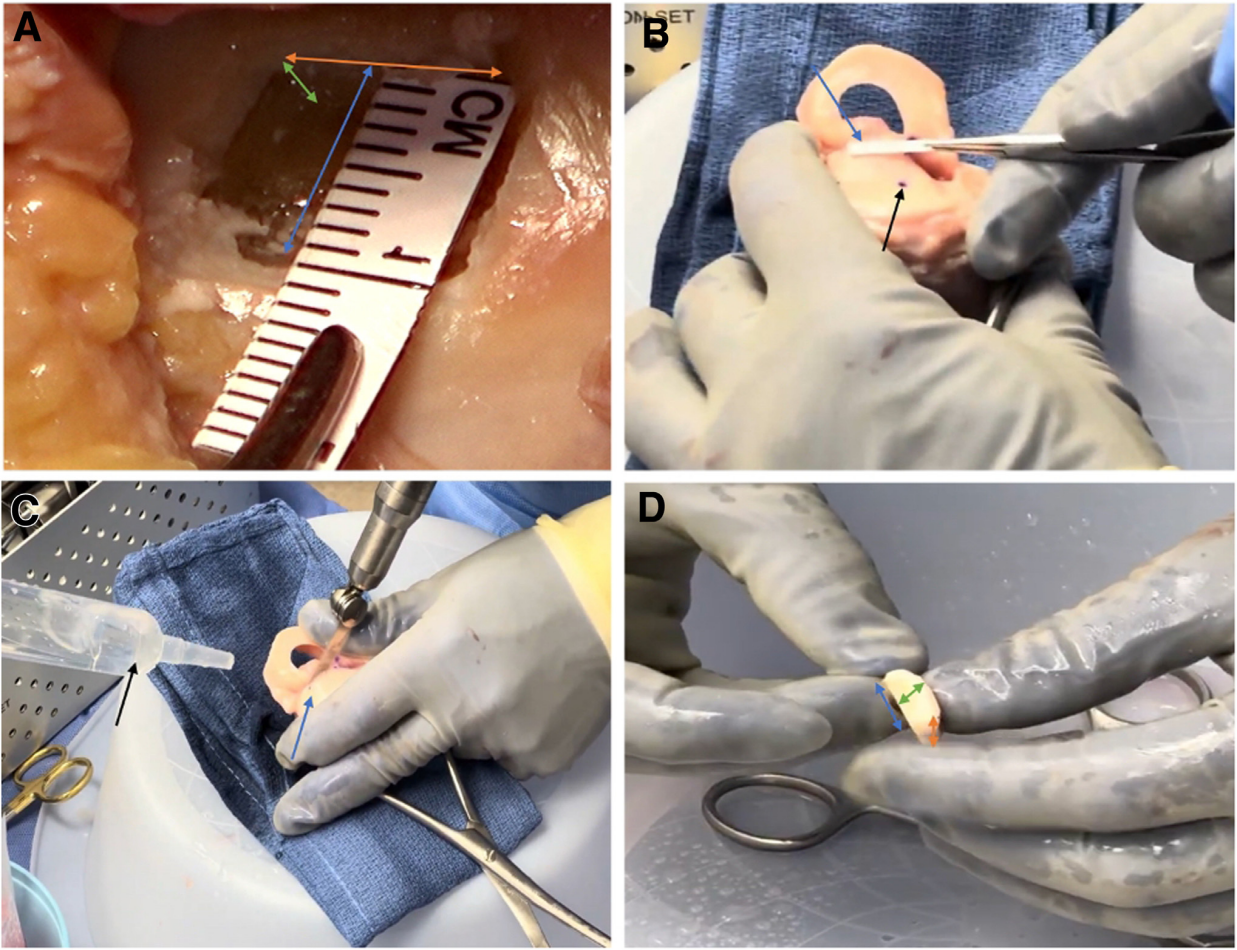
Osteochondral allograft preparation. (A) The defect of the right knee tibia plateau defect is measured with a ruler in length (blue double arrow), width (orange double arrow), and depth (green double arrow) under direct visualization through the lateral arthrotomy. (B) The dimensions are transferred to the donor right knee lateral tibia plateau by first marking the corresponding points (black arrow) and then applying the corresponding width and length (blue arrow). (C) The allograft plug is then cut out (blue arrow) under continuous irrigation (black arrow). (D) After the plug has been trimmed to the appropriate depth, the same dimensions are found as in the tibial defect (blue, green, orange double arrow).

The procedure begins with an arthroscopic evaluation to assess the lateral femorotibial compartment and to confirm the size and location of the tibial cartilage defect (Fig 1A). The lesion is debrided arthroscopically using a 3.5-mm shaver and curette, and the remaining unstable cartilage in the defect area is removed to enable thorough delineation and characterization. The arthroscopic procedure minimizes the risk of damage to the surrounding structures until a stable cartilage socket has been created. A lateral parapatellar arthrotomy is performed to expose the defect. Two Z-retractors are utilized to optimize visualization (Fig 1B). The first retractor is placed in the notch and is used to retract the extensor mechanism medially. The second retractor is placed along the lateral femoral condyle to retract the lateral capsule. The anterior horn of the lateral meniscus is detached for better visualization and access to the defect. With the knee in varus stress, the knee is flexed to 70°, and the tibia is internally rotated to bring the defect into view. The defect is then assessed under direct visualization, and additional debridement is performed if necessary. The damaged subchondral bone is then removed using a round burr (Fig 1C). The defect is chiseled out rectangularly until healthy, bleeding subchondral bone is observed, and stable vertical walls of surrounding healthy cartilage and bone are present (Fig 1D). A ruler is used to measure the depth of the defect in the 4 corners as well as the side length and width (Fig 2A).

Next, the focus shifts to the donor allograft. A matching orthotopic site is selected, typically harvested from the corresponding location to the patient’s defect. The size is marked according to the previous measurements, and the allograft plug is sawn out (Fig 2 B and C). Continuous irrigation is crucial to preserve cellular viability of the allograft. The graft is trimmed using a small oscillating saw to match the recipient site depth measurements (Fig 2D). Pulse lavage is applied to thoroughly remove bone marrow elements, reducing immunogenicity.

**Fig 3:**
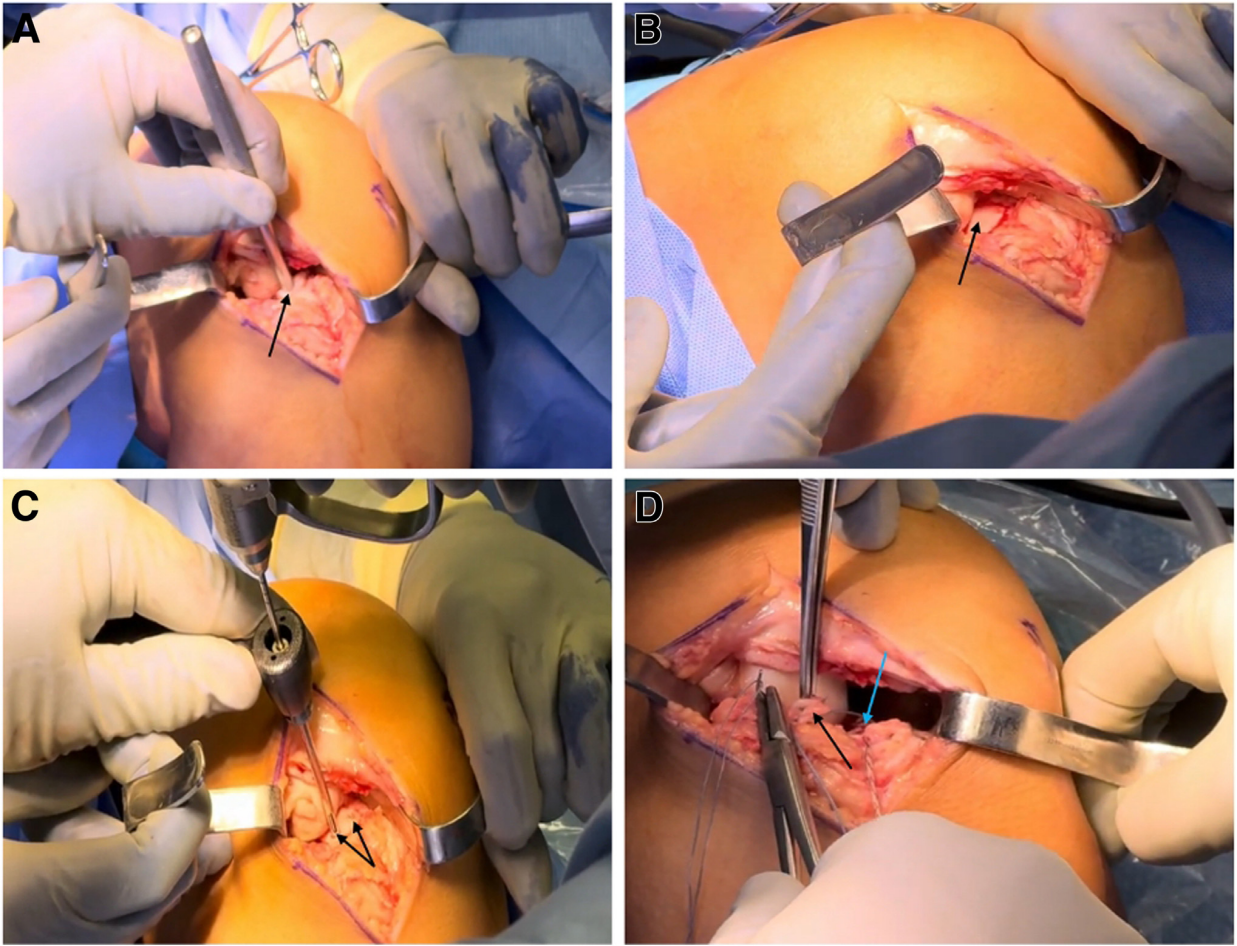
Osteochondral allograft implantation and fixation. (A) The allograft plug is press-fit into the recipient socket with gentle tamping over the right knee lateral tibia plateau (back arrow). During implantation the knee is in 70° flexion, varus stress and the tibia is rotated internally. (B) Appearance of the osteochondral allograft transplant in the lateral tibia plateau under direct visualization. (C) The plug is secured by 2 bioresorbable bone fixation nails (black arrows). (D) The detached anterior horn of the lateral meniscus (black arrow) is reattached to the original footprint (blue arrow).

Finally, the allograft plug is press-fit into the recipient socket, with gentle tamping over the TP to ensure the graft sits flush with the surrounding cartilage (Fig 3 A and B).

For further stabilization, the implant is fixed with 2 bioresorbable bone fixation nails (Fig 3C). For this purpose, a 1.5-mm drill is first used in the posterolateral and anteromedial region 20 mm through the fragment and into the solid bone. The bioresorbable bone fixation nails (SmartNail, 1.5 × 20 mm; ConMed) are then inserted so that they are just below the cartilage surface. The detached anterior horn of the lateral meniscus is then reattached to the original footprint using a 1.8-mm all-suture anchor (Fig 3D); the anchor (1.8 FiberTak Knotless Soft Anchor; Arthrex) is first positioned in the area of the footprint, and then the anterior horn is subsequently stitched and fixated using a free needle.

### Postoperative Rehabilitation

Postoperatively, patients are nonweightbearing for 4 weeks, then progress to weightbearing as tolerated, typically discontinuing crutches by week 6. A knee brace locked in full extension is worn for 2 weeks, then gradually opened by 20° per week between 2 and 4 weeks. At 6 weeks postoperatively, the knee brace is discontinued. Patients begin stationary biking, closed-chain exercises, and proprioception training around 4 weeks postoperatively, once 90° of flexion is obtained. Daily activities gradually resume at 3 months, beginning with nonimpact exercises between 3 and 6 months. Patients may begin jogging starting after 6 months, with full activity clearance at that time, although high-impact or knee-straining activities should be avoided for at least 8 months to protect surgical outcomes.^9^

## Discussion

The success of OCA implantation in managing isolated osteochondral defects of the patella or femoral condyles is well documented,^2^^,^^4^^,^^6^^,^^7^ while there remains limited reporting of outcomes in patients treated for tibial defects. The treatment of TP cartilage lesions poses unique challenges due to anatomic location, biomechanical loading, and limited surgical access. OCA transplantation is particularly suitable for large cartilage defects, osteochondral lesions, and revision procedures following failed previous cartilage surgery, notably when subchondral bone is damaged.^5^^,^^10^ Although data on the efficacy of OCA transplantation for focal cartilage lesions of the TP remain limited, a low rate of complications has been observed for surgical treatment of isolated TP cartilage lesions, with improved PROMs and 10-year survivorship ranging from 66.8% to 89%.^5^^,^^10^, ^11^, ^12^

Existing literature primarily focuses on hemi-tibial plateau allografts for larger unicompartimental defects, often secondary to trauma or complex knee pathologies.^5^^,^^10^, ^11^, ^12^ These approaches, while effective, are not necessarily ideal for isolated, central focal lesions without concomitant meniscus lesions or malalignment. Our technique addresses this gap by focusing on a targeted approach to treating central LTP defects using OCA transplantation. Patient selection, surgical planning, and technical precision are essential for the success of this procedure and to achieve favorable outcomes with OCA transplantation. The treatment of osteochondral defects of the TP presents a unique challenge, as the lesions are very individualized in shape and size. Standardized reamers cannot be used due to anatomic limitations.

Despite promising advances, challenges remain (Table 1). The lack of large high-quality studies on TP OCA limits the generalizability of outcome findings. Furthermore, the procedure’s complexity requires surgical expertise and careful patient selection (Table 2). Future research should focus on long-term outcomes and the development of standardized guidelines for TP cartilage restoration. In conclusion, OCA transplantation for focal LTP lesions represents a viable option for appropriately selected patients. Our described technique offers a targeted and effective approach to addressing these challenging defects while preserving knee function and structural integrity.

**Table 1:** Advantages and Disadvantages

Advantages	Disadvantages
Fresh allografts preserve chondrocyte viability, promoting better integration and healing. No donor site morbidity.	Limited availability of suitable donor grafts.
Lesions unsuitable for other procedures like autologous chondrocyte implantation or osteochondral autograft transplantation can be treated with osteochondral allograft implantation.	Technically demanding, requiring high surgical expertise. Limited standardization due to individualized defect characteristics and graft preparation.
May restore normal knee biomechanics, improving stability and movement.	
Low reported complication rates in isolated tibial plateau lesions.

**Table 2:** Pearls and Pitfalls

Pearls	Pitfalls
Carefully evaluate the patient’s knee pathology to ensure appropriate candidacy for the procedure.	For tibial cartilage defects requiring knee hyperflexion, the patient is positioned supine on a flat surgical table. Otherwise, a leg holder may be used to support the affected limb. Varus stress can improve access to lateral tibial defects.
Evaluate native alignment, which may prohibit indication or necessitate osteotomy.	Perform initial arthroscopic debridement to minimize soft tissue disruption and clearly demarcate the defect.
Fully evaluate extent of arthritic changes in all compartments.	Create a stable, rectangular cartilage socket with vertical walls for optimal graft fit. Application of additional fixation techniques enhances graft stability an integration.

## Disclosures

The authors declare the following financial interests/personal relationships which may be considered as potential competing interests: N.S. reports a relationship with *Arthroscopy* that includes editorial board membership. All other authors (S.S., C.B.G.L., D.F., A.B., O.P., C.L.) declare that they have no known competing financial interests or personal relationships that could have appeared to influence the work reported in this paper.

## Supplementary Data

Video 1 Osteochondral allograft transplantation for treatment of a focal lateral tibial plateau cartilage lesion.

## References

[1] Curl W.W., Krome J., Gordon E.S., Rushing J., Smith B.P., Poehling G.G. (1997). Cartilage injuries: A review of 31,516 knee arthroscopies. Arthroscopy.

[2] Chiang H., Jiang C.C. (2009). Repair of articular cartilage defects: Review and perspectives. J Formosan Med Assoc.

[3] Anderson C.J., Ziegler C.G., Wijdicks C.A., Engebretsen L., LaPrade R.F. (2012). Arthroscopically pertinent anatomy of the anterolateral and posteromedial bundles of the posterior cruciate ligament. J Bone Joint Surg.

[4] Gowd A.K., Weimer A.E., Rider D.E. (2021). Cartilage restoration for tibiofemoral bipolar lesions results in promising failure rates: A systematic review. Arthrosc Sports Med Rehabil.

[5] Melugin H.P., Bernard C.D., Camp C.L. (2019). Tibial plateau cartilage lesions: A systematic review of techniques, outcomes, and complications. Cartilage.

[6] Nassar J.E., Guerin G., Keel T. (published online November 4, 2024). Autologous chondrocyte implantation, matrix-induced autologous chondrocyte implantation, osteochondral autograft transplantation and osteochondral allograft improve knee function and pain with considerations for patient and cartilage defects characteristics: A systematic review and meta-analysis. Knee Surg Sports Traumatol Arthrosc.

[7] Ginesin E., Chari N.S., Barnhart J., Wojnowski N., Patel R.M. (2023). Cartilage restoration for isolated patellar chondral defects: An updated systematic review. Orthop J Sports Med.

[8] Bugbee W. (2018). Editorial Commentary: To treat or not to treat? Are we any closer to knowing what to do with cartilage lesions of the tibia?. Arthroscopy.

[9] Wagner K.R., Kaiser J.T., DeFroda S.F., Meeker Z.D., Cole B.J. (2022). Rehabilitation, restrictions, and return to sport after cartilage procedures. Arthrosc Sports Med Rehabil.

[10] Gracitelli G.C., Tirico L.E.P., McCauley J.C., Pulido P.A., Bugbee W.D. (2017). Fresh osteochondral allograft transplantation for fractures of the knee. Cartilage.

[11] Drexler M., Gross A., Dwyer T. (2015). Distal femoral varus osteotomy combined with tibial plateau fresh osteochondral allograft for post-traumatic osteoarthritis of the knee. Knee Surg Sports Traumatol Arthrosc.

[12] Shasha N., Krywulak S., Backstein D., Pressman A., Gross A.E. (2003). Long-term follow-up of fresh tibial osteochondral allografts for failed tibial plateau fractures. J Bone Joint Surg Am.

